# Sensory Modulation Disorder (SMD) and Pain: A New Perspective

**DOI:** 10.3389/fnint.2019.00027

**Published:** 2019-07-18

**Authors:** Tami Bar-Shalita, Yelena Granovsky, Shula Parush, Irit Weissman-Fogel

**Affiliations:** ^1^Department of Occupational Therapy, School of Health Professions, Sackler Faculty of Medicine, Tel Aviv University, Tel Aviv, Israel; ^2^Sagol School of Neuroscience, Tel Aviv University, Tel Aviv, Israel; ^3^Laboratory of Clinical Neurophysiology, Department of Neurology, Faculty of Medicine, Technion—Israel Institute of Technology, Rambam Health Care Campus, Haifa, Israel; ^4^School of Occupational Therapy, Faculty of Medicine of Hadassah, Hebrew University of Jerusalem, Jerusalem, Israel; ^5^Physical Therapy Department, Faculty of Social Welfare and Health Sciences, University of Haifa, Haifa, Israel

**Keywords:** sensory modulation disorder (SMD), pain perception and modulation, sensory over-responsivity (SOR), excitatory/inhibitory imbalance, sensory systems

## Abstract

Sensory modulation disorder (SMD) affects sensory processing across single or multiple sensory systems. The sensory over-responsivity (SOR) subtype of SMD is manifested clinically as a condition in which non-painful stimuli are perceived as abnormally irritating, unpleasant, or even painful. Moreover, SOR interferes with participation in daily routines and activities (Dunn, 2007; Bar-Shalita et al., 2008; Chien et al., 2016), co-occurs with daily pain hyper-sensitivity, and reduces quality of life due to bodily pain. Laboratory behavioral studies have confirmed abnormal pain perception, as demonstrated by hyperalgesia and an enhanced lingering painful sensation, in children and adults with SMD. Advanced quantitative sensory testing (QST) has revealed the mechanisms of altered pain processing in SOR whereby despite the existence of normal peripheral sensory processing, there is enhanced facilitation of pain-transmitting pathways along with preserved but delayed inhibitory pain modulation. These findings point to central nervous system (CNS) involvement as the underlying mechanism of pain hypersensitivity in SOR. Based on the mutual central processing of both non-painful and painful sensory stimuli, we suggest shared mechanisms such as cortical hyper-excitation, an excitatory-inhibitory neuronal imbalance, and sensory modulation alterations. This is supported by novel findings indicating that SOR is a risk factor and comorbidity of chronic non-neuropathic pain disorders. This is the first review to summarize current empirical knowledge investigating SMD and pain, a sensory modality not yet part of the official SMD realm. We propose a neurophysiological mechanism-based model for the interrelation between pain and SMD. Embracing the pain domain could significantly contribute to the understanding of this condition’s pathogenesis and how it manifests in daily life, as well as suggesting the basis for future potential mechanism-based therapies.

## A Pro-nociceptive State in Sensory Modulation Disorder (SMD)

Tactile over-responsiveness was characterized some decades ago as consisting of defensive-protective behaviors which are accompanied by stress responses to nociceptive qualities of sensory stimuli (Ayres, 1972; Fisher and Dunn, 1983). Specifically, non-painful sensory stimuli are often experienced by individuals with this disorder as aversive, bothersome (Kinnealey et al., 1995) and lingering (Miller et al., 2007). Despite these reports, the pain sensory system has been neglected in both the Sensory modulation disorder (SMD) clinical and research domains. Interestingly, *allodynia*, a clinical term not implying a mechanism, refers to pain due to a stimulus that does not normally provoke pain [International Association of the Study of Pain (IASP), 2017]. Consequently, allodynia represents a condition where the response mode differs from the stimulus mode [International Association of the Study of Pain (IASP), 2017], the latter of which may be induced by various non-painful stimuli such as light touch, cool or warm stimuli (Price, 1994; Zeilhofer, 2008). Therefore, we suggest allodynia to mirror sensory over-responsivity (SOR), a subtype of SMD, by perceiving non-painful sensations as irritating, unpleasant or painful (Miller et al., 2007). According to the International Association for the Study of Pain [International Association of the Study of Pain (IASP), 2017], pain is “an unpleasant sensory and emotional experience associated with actual or potential tissue damage or described in terms of such damage.” This definition of pain has led our research efforts for the past decade, where we have endeavored to further our understanding of the SOR phenomenon, by studying its phenotype as well as its underlying mechanisms.

Pain and other sensory systems are measured in the laboratory setting by performing quantitative sensory testing (QST), a standardized method to test for and characterize sensory sensitivity. QST measures the perceived intensity of a given stimulus (i.e., the subjective experience) while controlling the intensity of the stimulus (Dyck et al., 1993; McGrath and Brown, 2006; Hansson et al., 2007; Arendt-Nielsen and Yarnitsky, 2009). Moreover, it is used to indirectly evaluate the underlying sensory functioning by testing a spectrum of peripheral nerve system functions, as well as revealing abnormalities related to disorders of the central nervous system (CNS; Bartlett et al., 1998; Hagander et al., 2000; Arendt-Nielsen and Yarnitsky, 2009). Previous studies in our lab have used QST to evaluate somatosensory *detection thresholds* [i.e., the minimum intensity levels at which 50% of stimuli are recognized; International Association of the Study of Pain (IASP), 2017], including those of light touch, vibration, warm and cool sensations. We found no differences between individuals with SOR and those without, neither in children nor in adults. Furthermore, when measuring heat and cold *pain thresholds* [i.e., the minimum intensity levels of a stimulus that are perceived as painful; International Association of the Study of Pain (IASP), 2017], again, no such group differences were found (Bar-Shalita et al., 2009, 2012). In light of these findings, we showed that somatosensory detection and pain thresholds are not impacted in SOR. Intact sensory detection thresholds denote the absence of peripheral nerve system lesions. However, when we investigated laboratory-induced suprathreshold stimuli to measure the perceived pain intensity, we found group differences in both children and adults; individuals with SOR rated heat and mechanical painful stimuli as more painful than those without SMD, demonstrating hyperalgesia in the former group (Bar-Shalita et al., 2009, 2012; Weissman-Fogel et al., 2018). *Hyperalgesia* denotes abnormally increased pain from a stimulus that normally provokes pain, and like allodynia, it is a clinical term rather than a mechanism [International Association of the Study of Pain (IASP), 2017]. Furthermore, we revealed that in individuals with SOR the evoked pain sensation is higher in intensity and lingers for a longer duration after stimulus termination vs. non-SMD subjects who showed an expected gradual reduction in pain intensity that reached a level of no-pain within a 5–6 min time period (Bar-Shalita et al., 2009, 2012, 2014; Weissman-Fogel et al., 2018). This lingering sensation, termed *after-sensation*, validates the clinical symptoms reported by clients and could explain the accumulation of aversive sensations experienced by individuals with SMD throughout the day (Kinnealey et al., 2015).

After-sensation and hyperalgesia are both excitatory signs indicating central-sensitization that impacts pain perception (Andersen et al., 1996; Woolf and Salter, 2000; Woolf and Max, 2001; Gottrup et al., 2003; D’Mello and Dickenson, 2008). In SOR, we were the first to report the existence of a pro-nociceptive state resulting in pain amplification (Weissman-Fogel et al., 2018). Searching for this pro-nociceptive state underlying mechanism, we found inhibitory mechanisms which did not differ from non-SMD controls, though clearly presented a delayed process of inhibition. This emerged when testing the conditioned pain modulation (CPM) neurophysiological phenomenon, where one painful stimulus, the “conditioning stimulus,” inhibits a concomitant or subsequent painful “test stimulus” (Weissman-Fogel et al., 2018). Thus, individuals with SOR have central sensitization which is expressed as a pro-nociceptive state due to over excitation rather than reduced inhibition. Incoming sensory stimuli from the peripersonal space (“the spatial region surrounding the body that a person regards as theirs psychologically”; Senkowski et al., 2014) are experienced by an individual with SOR as painful (allodynia) and therefore require greater recruitment of top-down inhibitory mechanisms to support survival. In children and adults with SOR, their survival efforts are expressed by defensive-protecting behaviors when confronted with sensory stimuli intruding their peripersonal space. Indeed, quality of life is reduced in individuals with SOR, specifically due to bodily pain.

## Abnormal Central Sensory Processing in SMD

Current neurophysiological methods such as electroencephalography (EEG) have been used to define the neural origins of SMD. It has been found that the behavioral phenotype of SMD is due to atypical neural processing of both single *non-painful* sensory stimulus (i.e., somatosensory or auditory) and integration of simultaneous multi-sensory stimulation (i.e., somatosensory and auditory), This has been manifested by greater (Parush et al., 1997, 2007) and prolonged (Zlotnik et al., 2018) early event-related potentials (ERPs; a brain response to a specific external event) in response to tactile and auditory stimuli, respectively, along with smaller (Gavin et al., 2011) or greater (Davies et al., 2010) amplitudes of late auditory ERPs. This abnormally intense processing and lingering of sensory stimuli may result in individuals with SMD feeling overwhelmed when facing everyday sensory experiences. On top of this, adaptation deficiency to repetitive stimuli has been evident in ERPs (Kisley et al., 2004; Davies and Gavin, 2007; Brett-Green et al., 2010; i.e., ERP amplitude inhibition in response to repetitive paired-click stimulation), indicating a deficiency in pain inhibition probably due to an inefficient gating process. Moreover, atypical (neural integration of simultaneous multisensory stimulation (i.e., multisensory integration) has been indicated by spatio-temporal distribution of ERP responses to dual auditory and somatosensory stimuli (Brett-Green et al., 2010). Specifically, while in typically developing children multisensory integration occurs in central and post-central scalp regions during both early and later stages of sensory information processing (Brett-Green et al., 2008), those with SMD demonstrate a fronto-central distribution (Brett-Green et al., 2010). Accordingly, we have recently found (Granovsky et al., 2019) that subjects with SOR have different topographical dispersions of resting state EEG activity within the alpha band; while non-SMD individuals demonstrated increased activity toward parietal sites, those with SOR did not show this topographical distribution. Finally, novel findings from our lab point at an abnormal basic neurophysiological activity under a task-free condition in SOR individuals whereby there was a global reduction of cortical activity in theta, alpha and beta bands, most prominently in the alpha band, compared to non-SMD individuals. Thus, individuals with SOR demonstrate a neurophysiological state of a “non-resting” brain, which may partly explain their reported ongoing daily alertness to peripersonal stimuli. Furthermore, based on the “Gating by Inhibition” theory (Jensen and Mazaheri, 2010), alpha activity in higher-order cortical areas is mandatory for inhibiting task-irrelevant input. Thus, reduced alpha activity may consequently result in excessive sensory input processing which may contribute or result in SOR.

Studies have found associations between neurophysiological measures and behavioral manifestations of SMD, based on self- and caregiver reports of daily experience of sensory stimuli and functional performance on sensory tasks (Kisley et al., 2004; Gavin et al., 2011; Zlotnik et al., 2018). Namely, more sensory responsive or more avoiding behavior was correlated with higher amplitudes and more prolonged latencies of sensory response ERPs. This may reflect the major resources needed to process daily sensory stimuli among people with SMD. Moreover, such brain responses to sensory stimuli have correctly distinguished children with SMD from typically developing children and adults with 77%–96% accuracy (Davies and Gavin, 2007; Davies et al., 2010; Gavin et al., 2011). We, therefore, suggest that these neurophysiological differences may serve as characteristic markers of SMD that are underpinned by the anatomical abnormalities in sensory pathways (Owen et al., 2013) and which may contribute to the sensitive and/or avoidance behavior. This experience-induced neural plasticity may further mark its footprint in a sensory signature and thereby contribute to the sensory symptoms and daily life challenges experienced by individuals with SMD. Whether such a neurophysiological anomaly in individuals with SMD is nature or nurture, there is no doubt it reduces their successful social and functional participation in their home, school and community environments.

## An Excitatory/Inhibitory (E/I) Imbalance as a Shared Mechanism for SMD and Pain

The neurophysiological studies described above which investigated the central processes in response to external non-painful stimuli suggest an imbalance between excitatory and inhibitory processes in the brain. A balanced excitatory (glutamatergic) and inhibitory [γ-aminobutyric acid (GABA)ergic and glycinergic] ratio is essential for the brain to work appropriately in response to different sensory inputs. In adults, the tightly regulated E/I balance is achieved by homeostatic control of the strength and weight of transmissions in response to external stimuli. An increased E/I ratio can lead to a prolonged neocortical activity which may be associated with abnormal sensory processing such as hypersensitivity to different sensory stimuli (Zhang and Sun, 2011).

The E/I balance is one of the fundamental elements required for a normal sensory threshold and for regulating supra-threshold stimuli that originate from different sensory organs. In her early work, Ayres (Ayres, 1972) described the interrelationship of excitatory and inhibitory processes as modulation. Sufficient modulation occurs when the two processes work in harmony. Dunn (1997, 2001) developed a model of sensory modulation to explain the relationship between behavior and neurophysiological responses. Based on Dunn’s model of sensory processing, the nervous system’s functionality is represented by neurological thresholds whereby a “high threshold” requires a greater sensory input for activation while a “low threshold” requires lower stimulation for activation of sensory processing (Dunn, 1999). Behaviorally, individuals with low thresholds notice and respond to sensory stimuli more readily than the typical individuals, and thus represent a sensory profile that is sensory sensitive and sensory avoiding, defined as SOR (Miller et al., 2007). It is suggested by both Dunn (2001) and Miller et al. (2007) that individual sensory profiles are grouped based on psychophysiological measures, such as sensory thresholds and responses to supra-threshold stimuli, rather than by responses to specific sensory modalities. This, therefore, suggests that there are neurophysiological mechanisms common to more than one sensory system including the pain, auditory, tactile, and visual systems.

The hypersensitivity and lingering in response to experimental pain observed in individuals with SOR (Bar-Shalita et al., 2009, 2012, 2014; Weissman-Fogel et al., 2018) despite efficient habituation and inhibition capabilities (Weissman-Fogel et al., 2018) indicates increased neuronal excitation in the pain-transmitting pathways with no inhibition deficiency. We, therefore, suggest that the enhanced activity of pain-facilitatory pathways with preserved pain-inhibitory mechanisms in SMD may be related to an E/I imbalance (Weissman-Fogel et al., 2018). Glutamate, the main excitatory neurotransmitter, and GABA, the main inhibitory transmitter within the CNS play key roles in central pain processing. Specifically, glutamate plays an important role in pain transmission and modulation (see review: Goudet et al., 2009). The glutamate receptors are widely distributed throughout the CNS where they regulate cell excitability and synaptic transmission at different levels of the pain matrix. Expression of glutamate receptors have been reported in the thalamus (Lourenço Neto et al., 2000), amygdala (Neugebauer, 2007), and the midbrain periaqueductal gray region (PAG; Marabese et al., 2005) and generally serve a pro-nociceptive role (Goudet et al., 2009). The ascending dorsal horn nociceptive neurons project toward all these brain areas with the PAG being an important center for the processing of nociceptive information and descending modulatory circuitry. Glutamate receptors that have also been detected in glial cells which are active regulators and protectors of nervous system and therefore play a role in pain. On the other hand, GABA receptors have an important anti-nociceptive role in acute and chronic pain. At the supra-spinal level, they depress ascending adrenergic and dopaminergic input to the brainstem, and facilitate the descending noradrenergic input to the spinal cord dorsal horn (Goudet et al., 2009). Importantly, elevated brain glutamate levels (Harris et al., 2009; Prescot et al., 2009; Petrou et al., 2012) and lower levels of GABA (Foerster et al., 2012; Petrou et al., 2012) have been reported in chronic pain conditions. This neurotransmitter imbalance is manifested by neuronal hyperexcitability, which can be alleviated by anticonvulsants. Anticonvulsants inhibit neuronal hyperexcitability by multiple mechanisms including direct or indirect enhancement of inhibitory GABAergic neurotransmission, or inhibition of glutamatergic neurotransmission (Sullivan and Robinson, 2006).

The coupling between SOR to daily non-painful stimuli and enhanced pain facilitation suggests a common brain mechanism that is due to an E/I imbalance. This shared mechanism in SMD individuals who are pain-free may further serve as a predisposing factor for the development of pain disorders. Indeed, we recently found SMD to be a contributing factor for having complex regional pain syndrome (CRPS). CRPS is a chronic pain syndrome of unknown pathophysiology that develops after limb surgery or injury in 4%–7% of patients (Harden et al., 2010; Bruehl, 2015). Though the origin and progress of CRPS varies, it usually evokes a severe state of disablement in the affected limb, which robustly reduces function and quality of life (Lohnberg and Altmaier, 2013; van Velzen et al., 2014; Bean et al., 2016). No specific clinical sign or symptom has been found as a risk factor for CRPS onset (Pons et al., 2015). Yet, early identification of those at risk for CRPS is linked to enhanced outcomes (Li et al., 2010; Wertli et al., 2013). Our findings revealed that for a person with SMD the risk of CRPS is 2.68–8.21 times higher than for a person without SMD. Consequently, including the SMD domain as a risk factor in the CRPS clinical discussion prior to intervention may allow for an early diagnosis and a significant prognostic improvement.

## Multi-Sensory Processing Shaping the Pain Experience in SMD

Applying a nociceptive stimulus to the skin evokes activity imaged in a large network of brain regions which is referred to as the “pain matrix.” The pain matrix comprises the primary (S1) and secondary (S2) somatosensory cortices, the insula, and the anterior cingulate cortex (ACC; Treede et al., 1999; Peyron et al., 2002; Apkarian et al., 2005). However, Mouraux et al.’s (2011) findings challenge this model and suggest that the pain matrix regions are equally involved in processing non-nociceptive and nociceptive stimuli. Moreover, they postulate that most parts of the pain matrix are likely involved in cognitive brain processes that detect and process salient multisensory stimuli. Based on the hypothesis that most of the neocortex is multisensory (Ghazanfar and Schroeder, 2006), Senkowski et al. (2014) argue that pain-related neural responses at all processing stages can be shaped by non-painful stimuli. Different factors, such as stimulus intensity and valence, affect the way other sensory stimuli shape the pain perception. Specifically, painful stimuli accompanied by environmental input from other sensory modalities can impact not only the pain perception but also the processing of these stimuli. Other sensory modality stimuli may draw attention away and subsequently reduce the perceived pain intensity, or conversely, these stimuli can amplify the saliency of the painful stimuli and evoke an augmented pain experience. This suggests that non-painful stimuli in the peripersonal space have an important role in shaping the pain experience. Exploring this association, we found that the correlation between daily pain sensitivity and hyper-responsiveness tripled in individuals with SOR compared to non-SMD individuals (Bar-Shalita et al., 2015). Moreover, an unpleasant sensation intruding the peripersonal space usually evokes a defense response (Senkowski et al., 2014). Indeed, children and adults with SOR demonstrate and report protective responses to non-painful stimuli (Miller et al., 2007), which may be explained similarly to the main function of pain, warning of danger and preventing future tissue damage (Crombez et al., 2005; Dowman, 2011; Senkowski et al., 2014). Taken together, research on the multisensory shaping of pain has definite clinical implications (e.g., Senkowski and Heinz, 2016), but also offers an important novel understanding of the mechanisms as well as the relevance of multisensory processing to pain processing.

## Clinical Manifestation of SOR in Chronic Pain Conditions

Increased sensitivity to non-painful sensory stimuli is widely described for many chronic pain states. For example, in migraine, lower sensory thresholds, enhanced psychophysical and neurophysiological responses, and reduced adaptation and habituation to a specific sensory modality (usually visual or auditory) have all been reported including during the inter-ictal state (Harriott and Schwedt, 2014; Demarquay and Mauguière, 2016). Furthermore, many migraineurs report inter-ictal discomfort to everyday stimuli such as odors, light and sound, which may even trigger or worsen headache intensity (Vanagaite et al., 1997; Martin et al., 2006; Borini et al., 2008; Friedman and De Ver Dye, 2009; Noseda and Burstein, 2013; Schwedt, 2013). Thus, this multi-sensory hypersensitivity may point to an abnormal central multisensory integration in migraine (Schwedt, 2013).

Similar to the suggested SMD pathophysiology, the underlying neurophysiological mechanisms of increased sensitivity in inter-ictal migraine suggest alterations in the cortical circuits and neurotransmitters which maintain the E/I balance (Pietrobon and Moskowitz, 2013; Demarquay and Mauguière, 2016). Moreover, the results of our recent study have revealed that 45% of migraine patients are diagnosed with SMD (Granovsky et al., 2018), an incidence far above the ~10% SMD incidence (range 5%–16%) among pain-free healthy pediatric and adult populations (Ahn et al., 2004; Ben-Sasson et al., 2009; Bar-Shalita et al., 2015). The association of SOR with migraine pain symptoms such as having sensory aura, a higher frequency of monthly attacks, and an enhanced activity of pain facilitatory pathways (Granovsky et al., 2018) further support the inter-relation between non-painful sensory and pain transmitting pathways (Schwedt, 2013). An example of this is a study reporting that experimentally-evoked trigeminal pain further enhances the cortical hyperexcitability and the lack of habituation to light in migraine patients (Boulloche et al., 2010). This phenomenon can be related to the anatomical integration of pain and visual processing in thalamic nuclei (Noseda and Burstein, 2013) that project to cortical areas involved in the processing of pain and visual perception. We can only hypothesize about a similarity of the central neuroanatomical integration alterations in sensory and pain-transmitting pathways to that described in migraine.

Another chronic pain state characterized by a global disturbance in sensory responsiveness is fibromyalgia (FM). Many studies have reported on greater sensitivity to various non-painful sensory experimental stimuli (tactile, thermal, electrical, auditory) in FM (Lautenbacher et al., 1994; Montoya et al., 2006; Geisser et al., 2008; Hollins et al., 2009). Similar to migraine, FM patients have also enhanced sensory responses to everyday real-life stimuli such as auditory stimuli (Geisser et al., 2008) and cutaneous sensations (Borg et al., 2015). This greater sensitivity is known as a “generalized hypervigilance” and is considered as one of the pathophysiological mechanisms of FM (McDermid et al., 1996; Rollman, 2009). Some authors also refer to heightened affective, sensory and pain responses as an abnormality of the interoceptive system in FM (Lovero et al., 2009; Seth and Friston, 2016; Duschek et al., 2017; Valenzuela-Moguillansky et al., 2017; Martínez et al., 2018). Along with the widely reported pro-nociceptive pattern of psychophysical and neurophysiological responses (Staud and Spaeth, 2008; Staud, 2011; O’Brien et al., 2018), sensory over-responsiveness in FM can point to a decrease in inhibitory and/or an increase in facilitatory activity in the CNS.

Since pain is a multidimensional and complex experience composed of sensory, affective-motivational, cognitive-evaluative components (Melzack and Casey, 1968), we propose the SMD as another factor that may shape the pain experience.

## Abnormal EEG Responses as a Shared Mechanism for SMD and Pain

In migraine and FM, along with enhanced pain psychophysical responses, cortical activity has been repeatedly shown to be abnormal. More specifically, reports from many studies have pointed to higher amplitudes of early (A-delta mediated) pain-evoked ERPs (Gibson et al., 1994; Lorenz et al., 1996; Lev et al., 2010; de Tommaso et al., 2011; Truini et al., 2015), along with deficient habituation of these and other neurophysiological responses (Valeriani et al., 2003; Lev et al., 2010; de Tommaso et al., 2014, 2015; Harriott and Schwedt, 2014). Similarly, research in SMD has also indicated higher (Parush et al., 1997, 2007) and prolonged (Zlotnik et al., 2018) early ERPs in response to non-painful sensory stimuli along with an adaptation deficiency (Kisley et al., 2004; Davies and Gavin, 2007; Brett-Green et al., 2010). These neurophysiological markers again suggest a shared mechanism in SMD and chronic pain, namely, enhanced cortical activity and deficient inhibition.

Though brain imaging studies in SMD are yet to come, we can deduce from a standardized low resolution brain electromagnetic tomography (sLORETA) study in migraine that these neurophysiological markers may be linked with enhanced activity of S1 and reduced activity of the orbitofrontal cortex (the part of the prefrontal cortex associated with initiation of pain inhibition; Lev et al., 2010). In migraine, these neurophysiological activity patterns are observed in painful as well as non-painful stimuli (de Tommaso et al., 2013) and moreover are correlated with the clinical characteristics (Lev et al., 2013).

An abnormal pattern of EEG responses in chronic pain patients is also reported in resting-state conditions. The most consistent reported findings refer to the abnormal alpha, theta or beta activity in migraine and FM. More specifically in migraine, increased alpha power has been recorded in posterior brain regions, while activity in the frontal lobe has revealed decreased activity in alpha generators (Clemens et al., 2008; Cao et al., 2018). Other studies have also reported on a global inter-ictal decrease of EEG activity (Tsounis and Varfis, 1992; Cao et al., 2016) and on an association between slower alpha activity and greater disease and attack durations (Bjørk et al., 2009). Whereas in FM, decreased alpha, increased beta (Vanneste et al., 2017) and augmented theta activity (Fallon et al., 2018) have been found in different cortical areas and have also been reported to positively correlate with clinical symptoms. Interestingly, abnormal alpha activity and a global reduction of cortical activity in theta, alpha and beta bands has also been observed in SMD (Granovsky et al., 2019).

Further validation for the suggested link between chronic pain and SMD is evident in our recent unpublished data on migraineurs (article in preparation). Our research has indicated that lower connectivity values in the theta band at centro-parietal region are correlated with higher scores in SOR.

## Sensory Modulation Alterations as a Shared Mechanism for Chronic Pain and SMD

The assessment of pain modulation is performed by using various stimulation protocols which include a combination of different stimulus modalities and psychophysical tests. The latter selectively engage the pain facilitatory bottom-up or pain inhibitory top-down pathways and are believed to reflect the “real-life” modulation process exerted by patients when exposed to clinical pain. One of the most studied mechanisms of the supraspinally-mediated descending pain inhibitory system is the diffuse noxious inhibitory control (DNIC). DNIC engages the activation of the endogenous analgesia system, where upon arrival of data to the brainstem the ascending pain activates descending pain inhibitory pathways, exerting effects on incoming nociceptive inputs (Le Bars, 2002). The pain alleviating efficiency of DNIC relates on the balance between the anti-nociceptive effect of noradrenergic neurotransmission, and pro- or anti-nociceptive effect of serotonergic neurotransmission, that depends on the type of serotonin receptor (Bannister and Dickenson, 2016). The neurophysiological mechanism for the activation of bottom-up facilitatory pathways is associated with the glutamate-mediated windup of second-order neurons and reflects the state of central neuronal sensitization (Woolf and Thompson, 1991). Moreover, imbalance between the excited pain facilitatory systems, and the reduced activity in pain inhibitory pathways, including reduced functional connectivity with the brain regions associated with pain inhibition and/or enhanced connectivity with the brain regions associated with pain facilitation (Wang et al., 2016; Harper et al., 2018) point on a pro-nociceptive pain modulation profile as reported in many chronic pain states (Granovsky and Yarnitsky, 2013; Yarnitsky et al., 2014; Yarnitsky, 2015), including migraine and FM. Despite the still open chicken-and-egg question on the causality of the interrelations between the modulation state and the presence of the various pain syndromes, it is believed that a pre-existing facilitatory state of the CNS leads to the establishment of a pro-nociceptive profile and the acquisition of chronic pain syndromes. This causative relation was found in a longitudinal study on pain-free pre-thoracotomy patients, demonstrating that those with less-efficient endogenous pain inhibition had a higher incidence and intensity of chronic post-operative pain (Yarnitsky et al., 2008). These results were later reproduced for cesarean section and major abdominal surgery patients, respectively (Landau et al., 2010; Wilder-Smith et al., 2010). All the above findings taken together demonstrate that SMD is a pro-nociceptive condition (Weissman-Fogel et al., 2018). We propose that SOR is a predisposing factor or risk factor for chronic pain.

## Summary

**Figure 1:**
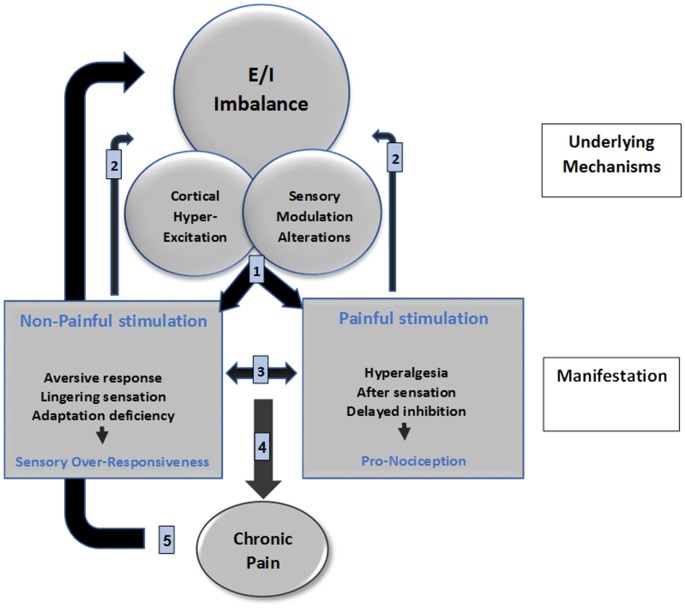
SMDolor model depicts a neurophysiological mechanism-based model for the interrelation between pain and SMD. The numbers represents the putative processes that manifest clinically: SOR, and pro-nociceptive state expressions of central alteration (1); both conditions elicit stimuli processing impact on brain mechanisms due to brain plasticity (2); and also create a bi-directional impact on the sensory perception (3); which may accumulate to develop chronic pain as a consequence of pro-nociception (4); which then nurtures the brain mechanisms alterations via brain plasticity (5).

We propose a neurophysiological mechanism-based model for the interrelation between pain and SMD, namely the SMDolor Model (Figure 1; the numbers guide the following explanation). Shared central neural mechanisms between SOR and pain, E/I imbalance; cortical hyper-excitation and sensory modulation alterations, are the cornerstone of this proposed model. These shared mechanisms are behaviorally expressed (1) as SOR in sensory systems processing non-painful stimuli, and as a pro-nociceptive state when processing painful stimuli. Daily life events require a multi-sensory integration for adaptive responding. This warrants a convergence of sensory stimuli from different modalities including pain which in turn causes pain to be influenced by these other sensory stimuli and vice versa (3), consequently, daily life events are experienced as aversive, irritating, and painful by individuals with SOR. These experiences induce neuronal plasticity (2) that may further result in a sensory signature which strengthens the abnormal shared mechanisms, contributing to the sensory symptoms that shape the daily life challenges experienced by individuals with SOR. These loop reactions may in some cases accumulate up to the point of developing a chronic pain condition (4). Chronic pain may then further nurture the shared central neural mechanisms (5).

## Author Contributions

All authors listed have made a substantial, direct and intellectual contribution to the work, and approved it for publication.

## Conflict of Interest Statement

The authors declare that the research was conducted in the absence of any commercial or financial relationships that could be construed as a potential conflict of interest.
